# Bright Light as a Personalized Precision Treatment of Mood Disorders

**DOI:** 10.3389/fpsyt.2019.00085

**Published:** 2019-03-01

**Authors:** Julia Maruani, Pierre Alexis Geoffroy

**Affiliations:** ^1^Inserm, U1144, Paris, France; ^2^Université Paris Descartes, UMR-S 1144, Paris, France; ^3^Université Paris Diderot, Sorbonne Paris Cité, UMR-S 1144, Paris, France; ^4^AP-HP, GH Saint-Louis–Lariboisière–F. Widal, Pôle de Psychiatrie et de Médecine Addictologique, Paris, France

**Keywords:** bright light therapy, circadian rhythms, sleep, seasonal affective disorder, non-seasonal depression, bipolar disorder

## Abstract

**Background:** The use of light for its antidepressant action dates back to the beginnings of civilization. Three decades ago, the use of bright-light therapy (BLT) for treating Seasonal Affective Disorder (SAD) was officially proposed. Since then, a growing scientific literature reports its antidepressant efficacy in both unipolar and bipolar disorders (BD), with or without seasonal patterns. This review aims to examine the management of BLT as a personalized and precision treatment in SAD, unipolar, and BD.

**Methods:** We conducted a narrative review using Medline and Google Scholar databases up to June 2018.

**Results:** BLT has physiological effects by resynchronizing the biological clock (circadian system), enhancing alertness, increasing sleep pressure (homeostatic system), and acting on serotonin, and other monoaminergic pathways. Effects of BLT on mood depend on several factors such as light intensity, wavelength spectrum, illumination duration, time of the day, and individual circadian rhythms. A growing body of evidence has been generated over the last decade about BLT evolving as an effective depression treatment not only to be used in SAD, but also in non-seasonal depression, with efficiency comparable to fluoxetine, and possibly more robust in patients with BD. The antidepressant action of BLT is fast (within 1-week) and safe, with the need in BD to protect against manic switch with mood stabilizers. Side effects might be nausea, diarrhea, headache, and eye irritation, and are generally mild and rare. This good safety profile may be of particular interest, especially in women during the perinatal period or for the elderly. The management of BLT needs to be clarified across mood disorders and future studies are expected to compare different dose-titration protocols, to validate its use as a maintenance treatment, and also to identify predictive biomarkers of response and tolerability. We propose clinical guidelines for BLT use in SAD, non-seasonal depression, and BD.

**Conclusions :** BLT is an efficient antidepressant strategy in mono- or adjunct-therapy, that should be personalized according the unipolar or bipolar subtype, the presence or absence of seasonal patterns, and also regarding its efficacy and tolerability.

## Introduction

The use of light for its antidepressant action dates back to the beginnings of civilization (1). Three decades ago, the use of bright-light therapy (BLT) for treating Seasonal Affective Disorder (SAD) was officially proposed. It is now acknowledged as an antidepressant strategy for mood disorders (2–4). In the 1980s, BLT was developed in SAD to extend daytime photoperiod and counteract winter darkness (5). BLT is now considered to be the first line treatment for SAD in therapeutic guidelines (2). Since then, a growing scientific literature reports its antidepressant efficacy in both unipolar and bipolar disorders (BD), without such seasonal patterns. Indeed, the sustained antidepressant efficacy of BLT, used alone or in combination with antidepressant drugs–but also with some mood stabilizers and sleep deprivation, has been evidenced in numerous clinical studies (2, 6, 7). This antidepressant effect may be both due to light's effect on the biological clock -by phase advance and alignment of circadian rhythms- and/or actions on non-circadian pathways (8). Indeed, light modulates the activation of efferent serotonergic neurons, decreases the serotonin reuptake transporter (5-HTT) levels, and increases serotonin (5-HT) levels in mood regulatory areas such as the anterior cingulate and prefrontal Cortex (1, 9). Recent reviews discuss how light may influence mood, and emphasize recent finding of light's direct effects on enhancing alertness and the sleep homeostasis (10). Thus, light exerts strong effects on mood thanks to many circadian and non-circadian actions that may combine: phase shifting of circadian rhythms, enhancement alertness, sleep homeostasis by increasing EEG delta activity and sleep pressure, and modulation of the serotonin and other monoaminergic pathways. These effects of BLT on mood depend on several factors such as light intensity, wavelength spectrum, illumination duration, time of the day, and individual circadian rhythms (3).

However, the management of BLT continues to be a point of debate in mood disorders, with no evidence-based guidelines for implementing BLT in patients across mood disorders (2, 11, 12). This review aims to examine the management of BLT as a personalized and precision mood disorders treatment, encompassing both unipolar and bipolar disorders, and seasonal and non-seasonal characteristics.

## Materials and Methods

### Search Strategy

We aimed to consider papers examining efficacy of BLT in mood disorder including SAD, unipolar and bipolar disorders, with or without seasonal characteristics. Only data published in English and French were included in this review. We conducted a narrative review using Medline and Google Scholar databases up to June 2018, using the following keywords combination: (“depression” or “bipolar disorder” or “unipolar disorder,” or “seasonal affective disorder”) and (“light therapy” or “phototherapy”).

### Study Selection

Two authors (JM, PAG) reviewed the title and abstract of identified publications in order to identify eligible studies. The two resulting article lists were compared and, in case of disagreement, the final decision as to inclusion was made by consensus. JM and PG independently and then jointly selected studies for detailed extraction of information, mostly based on the full text. In cases where full text was not available, corresponding authors were contacted. If a reply was not obtained following a 6-month waiting period, abstracts were then considered in the review only if the appropriate information was included. The exclusion criteria included reviews, meta-analyses, commentaries, case reports, and studies where bright light therapy on patients with BD or SAD or unipolar depression was not investigated. Finally, we decided to divide literature results in four main sections: (1) BLT in SAD; (2) BLT in non-seasonal depression; (3) BLT in BD depression; (4) BLT for sleep and circadian rhythms abnormalities associated in chronic mood disorders.

## Results

The literature search returned 234 records pertaining to BLT, SAD, and unipolar and bipolar disorders, with or without seasonal characteristics. Following preliminary screening of the titles and abstracts, 125 records were excluded (reviews, meta-analyses, commentaries, case reports, and studies where bright light therapy on patients with BD or SAD or unipolar depression was not investigated). With 41 studies identified from the related articles function of the PubMed database and the reference list of retained studies, 84 independent studies were retained in the qualitative analysis. These investigations were classified according to their studied parameters: (1) 17 Studies explored BLT in SAD; (2) 40 studies explored BLT in non-seasonal depression; (3) 12 studies explored BLT in BD depression; (4) 15 studies explored BLT for sleep and circadian rhythms abnormalities associated in chronic mood disorders.

### Bright Light Therapy in Seasonal Affective Disorders (SAD)

#### BLT as an Effective Curative Treatment in SAD

Among mood disorders, seasonal affective disorder (SAD) corresponds to the seasonal pattern of recurrent major depressive episodes occurring during the same time of the year, usually in autumn or winter with spontaneous remission in the spring or summer (5). This disorder is frequent, with prevalence varying between 0.4 and 16% in the general population according to latitude, age, sex, and the method of measurement used (13). SAD is a severe transdiagnostic disorder that may affect patients with both unipolar and bipolar disorders (5, 14). Over the past two decades, researchers, and clinicians have mainly focused on the pathophysiological mechanisms involved in SAD. Studies support the existence of interactions between a genetic vulnerability and chronobiological factors, and brain process alterations including noradrenergic and serotoninergic neurotransmissions (8). Several international therapeutic guidelines and many studies suggest that BLT is a non-pharmacological antidepressant that has proved to be effective in SAD and is now used as the first line treatment for individuals with SAD because of its low side effects profile and high response rate about 67% in patients with milder SAD and 40% in severe SAD patients (2–4, 15–17).

#### Usage in SAD

BLT is classically delivered through a light box that is equipped with fluorescent tubes and a reflector or diffusing screen. Patients sit in front of the light box mounted on a table with their eyes open. BLT may be also administered thanks to light glasses or visors (18, 19).

Treatment in SAD may begin with exposition duration of 30 min, using a light intensity of 10,000 lux. Early morning administration offers greater chances for remission (20, 21). Measured at eye level, a therapeutic distance of 60–80 cm from the light box can be seen as standard requirements (some other devices recommend a distance of 30 cm, so we advise to follow the device recommendations that take into account light parameters and distance). Lower intensities also appear to be effective, but need longer exposure durations: 2,500 Lux for 2 h/day, 5,000 Lux for 1 h/day. Significant effects appear only at 2–3 weeks of treatment. Treatment is usually continued until the time of usual spontaneous remission in the spring or summer because the effects of LT do not persist after discontinuation of BLT. In addition, it has been observed that low-intensity blue-enriched light has a therapeutic effect comparable to standard bright light (10,000 lux) in treating SAD (22). Finally, BLT is well-tolerated by patients; adverse effects such as headache, eyestrain, nausea and agitation, are usually transient and mild (23, 24). Main contraindications are ophthalmic disorders (cataract, macular degeneration, glaucoma, retinitis pigmentosa) and disorders affecting the retina (retinopathy, diabetes, herpes, etc.); and patients at risk (or if there is a doubt) should have pretreatment ophthalmological examinations (3).

#### BLT as a Preventive Device for Seasonal Affective Disorder

Patients with SAD might benefit from prophylactic use of BLT (25, 26). A recent Cochrane review assessed the efficacy of BLT in preventing SAD (27). Both forms of preventive light therapy (light boxes and visor) reduced the incidence of SAD compared with no light therapy. Although not statistically significant, they observed that BLT reduced the risk of SAD incidence of 36% (27). However, given methodological limitations (small sample sizes of available RCTs, and lack of power for some analyses), authors concluded that the decision for or against initiating preventive treatment of SAD and the treatment selected should be strongly based on patient preferences.

### Bright Light Therapy for Non-seasonal Depression

#### BLT as an Effective Treatment in Non-seasonal Depression

In the last two decades, the interest in BLT has expanded far beyond SAD. Indeed, several studies investigated the efficacy of BLT in treating non-seasonal depression disorders as alternative or adjunctive treatment. This is of major interest because depression affects an estimated 350 million people worldwide and is projected to become the second global leading cause of disability by the year 2020 (28). Moreover, only 50–60% of patients respond to first line antidepressants and only 35–40% experience remission of symptoms (29). Last but not least, pharmacological antidepressant strategies as first line treatments take at least 4 weeks to build up its effect and work fully (11).

Recent systematic reviews and meta-analyses confirmed this extent of antidepressant efficacy in non-seasonal depressions. Indeed the APA Committee on Research on Psychiatric treatment (2) and a Cochrane review (12) observed significant effect sizes equivalent to those in most antidepressant pharmacotherapy trials that were about 0.84. Reports and double blind placebo controlled studies suggest that the efficacy of BLT as an adjunct therapy in treating non-seasonal depression in its initial phase is faster and is perceived during the first week of treatment (7, 30, 31). These studies also confirmed the efficacy of the combination of BLT and selective serotonin reuptake inhibitors (SSRIs) with benefit after 1 month of treatment. BLT combined with SSRI lead to a faster (within a week) and better remission of patients (by reducing 30% of symptoms) in patients with non-seasonal depression than SSRIs alone (7, 31). In line with this, results are confirmed in a recent meta-analysis that also conclude that BLT are effective for patients with non-seasonal depression with clinical significant effect (SMD = −0.62, *P* < 0.001, I^2^ = 37%) and can be a helpful additional treatment for depression (32). This meta-analysis included 419 patients with non-seasonal depression (unipolar or bipolar depression) from 9 trials: 211 receiving BLT and 208-placebo controls. Most participants received BLT as a monotherapy except in two trials where they had BLT in addition to antidepressant. First, their results reported significant effects in the first week when administered in the early morning (2, 12). They found the largest antidepressant effect of BLT for an exposure duration of 2–5 weeks, and unfortunately were not able to propose an optimal intensity of BLT given the heterogeneity of the trials. Another recent meta-analysis of randomized controlled trials (11), including 881 participants from 20 RCTs used BLT as monotherapy compared to an inactive placebo/control group; and also as an adjunctive treatment in comparison to the same control group. They considered individuals with all depression subtypes excepting SAD: major depressive disorder; persistent depressive disorder, and BD depression. All forms of BLT (timing of administration, brightness, and duration of light exposure) were included, even though most studies (*n* = 5) used bright white light at 10,000 lux in early morning for 30 min/day or 2,500 lux for 120 min/day. They found that BLT was associated with a small to moderate effect in reducing symptoms in adults (11). Interestingly, studies demonstrated twice the reduction in depressive symptoms for BLT than placebo. Moreover, meta-analyses of Perera et al. (11) and Golden et al. (2) both found that BLT may be most effective when applied as an antidepressant monotherapy (and not as an adjunct treatment), when administered in the morning and among out-patients, that may have less severe depressive symptoms and comorbidities than in-patients. So, taken as a whole, patients who are non-responsive or ineligible for pharmacotherapy may benefit from monotherapy BLT, but BLT could also be considered as an effective first line treatment. For the elderly, BLT also seems to be efficient (33–35). Recently a systematic review in non-seasonal geriatric depression found that BLT during 6 weeks, with exposure duration varying between 30 and 60 min and light intensity varying from 1,200 to 10,000 Lux, is an effective treatment for reducing the depression symptoms in the elderly (34).

Finally, regarding safety in non-seasonal depression, side effects are rare and generally mild: nausea, diarrhea, headache, and eye irritation (11). This good safety profile may be of particular interest for women with depression in the perinatal period where medications may be inappropriate or ineffective, and also in the elderly.

#### Usage in Non-seasonal Depression

Precise recommendations regarding the optimal treatment (i.e., optimal exposure duration and intensity) are difficult because of the heterogeneity of study protocols and absence of comparisions between protocols (32). However, it is possible to say that BLT is confirmed to be efficient both as a mono- or adjunct-therapy in treating non-seasonal depression in his acute phase, with benefits that can be perceived during the first week of treatment. The effects of BLT do not appear to persist after discontinuation with a complete offset of effect after 1 month (36), and this relapse can be prevented when combining BLT with common antidepressant drugs (37). According to previous studies, daily early morning exposures to 2,500 Lux for 2 h (38), 5,000 Lux for 1 h (39), or 10,000 Lux for 30 min (40) all appear efficient in reducing antidepressant symptoms. Finally, BLT is well-tolerated by patients, and possible adverse effects might be headache, eyestrain, nausea, and agitation, that are usually transient and mild (see Table 1).

**Table 1:** Forms of bright light therapy use in different mood disorders.

	**Seasonal affective disorder (SAD)**	**Non-seasonal unipolar disorder**	**Non-seasonal bipolar disorder**
Light intensity	2,500–10,000 Lux Or low-intensity blue-enriched light	2,500–10,000 Lux	<10,000 Lux
Dose (Exposure durations/Intensity of illumination)	First line: 10,000 Lux for 30 min/day Also consider: 5,000 Lux for 1 h/day or 2,500 Lux for 2 h/day		Slower increase For instance: 5,000 Lux with increase of 15 min per week until 60 min at one month (depending on efficacy and tolerance)
Lamp disposition	lamp at eye level distance of 30–80 cm (depending on the device recommendations)		
Frequency Administration	Daily Mono- or adjunct-therapy		Daily Only with a mood stabilizer with antimanic properties
Time of the day	Early morning (for instance: 8 am, chronotype may be considered)		Midday (especially if there is an history of manic switch) Or Early morning
Onset of response	1 week		
Duration of treatment	Until the period of usual spontaneous remission in the spring or summer	2–5 weeks	Until reduction of depressive symptoms or maintained in case of relapse when stopped
Prevention	Possibility to treat by light therapy a few weeks before the usual seasonal depressive relapse period	NA	NA
Adverse effects	Manic switch and mild side effects (headache, eyestrain, nausea, and agitation)	Mild side effects (headache, eyestrain, nausea, diarrhea, and agitation)	Manic switch and mild side effects (headache, eyestrain, nausea, and agitation)
Contraindications	Ophthalmic disorders (cataract, macular degeneration, glaucoma, retinitis pigmentosa), and disorders affecting the retina (retinopathy, diabetes, herpes, etc.)		

*NA, data not available*.

### Bright Light Therapy in Acute Bipolar Disorder Depression

#### BLT as an Effective Treatment in Bipolar Disorder Depression

About 1–4% of the worldwide population suffers from bipolar disorder (BD), which is a severe mental disorder associated with both depressive and manic episodes that may be induced by antidepressants (41, 42). Given the limited treatment options in BD depression, since Lewy's study (43) several researches have focused on investigating BLT in this particular population because BD are increasingly recognized as disorders of the biological clock (44, 45), with circadian dysregulation being evident in both acute and remission phases (46, 47). Indeed, research has shown that patients with BD depression responded robustly to BLT (1). While the efficacy of BLT in monotherapy is non-significant in some studies (48, 49), the combination of BLT with other chronotherapeutic techniques such as sleep deprivation and with lithium salts was proven in BD depression patients (6, 50).

First, Leibenluft et al. showed that BLT at midday could be tailored to counteract depressive swings without exacerbating mania, in course of rapid cycling BD (51). Later, Benedetti et al. showed that morning sunlight reduces length of hospitalization in BD depression by comparing a sample of 415 unipolar and 187 bipolar depression inpatients assigned with eastern or western windows (52). They found that inpatients in eastern rooms exposed to direct sunlight in the morning had a shorter hospitalization than patients in western rooms (52). In 2005, the same team showed that combination of total sleep deprivation and BLT in drug-resistant patients with BD depression was useful in triggering an acute response (6). Since 2005, several randomized controlled studies and meta-analyses of randomized controlled studies focused on BLT efficacy in BD and confirmed that BLT is an effective and safe option as an adjunctive therapy in BD depression (53–56). Indeed, Yorguner Kupeli et al. confirmed in a randomized single blind placebo-controlled study the efficacy of BLT as an add-on treatment for BD depression when it is administered in the mornings at 10,000 lux for 30 min for a 2-week period, sitting 40–70 cm's away from the device (56). Zhou et al. also confirmed in a randomized single blind placebo controlled study the efficacy of 1 h, 5,000 lux, every morning -between 6:30 am and 9 am- of BLT as an add-on therapy, by reduction of depressive symptoms and observed onset efficacy at 4 days (54). Interestingly, no participants experienced symptoms of mania and no serious adverse effects were reported. Regarding midday BLT, Sit et al. performed a 6 week randomized double–blind placebo-controlled trial and found results supporting midday BLT as an efficient therapy in BD depression (55). In this RCT study, light therapy was administered as an add-on therapy (anti-manic or antidepressant medication) to patients with a current moderate or severe depression and showed that the BLT group (7,000 Lux at midday) had significantly higher remission rates (56%) vs. the 50-lux dim red light (14.3%). Duration of light therapy was increased progressively every week by 15 min to attain a target dose of 60 min per day at 4 weeks (55). Again, in this randomized controlled trial, no hypomanic/manic shift was observed, contrary to a previous report from the same team that observed (hypo)manic switches in females with rapid cycling BD and morning exposures (55). Benedetti (57) performed a systematic review of the literature studies reporting effect of antidepressant BLT in BD and their conclusions were limited again by the heterogeneity of treatment modalities between studies. Nevertheless, their review managed to include 799 treated patients and shows that the rate of switch into mania after morning BLT was small and close to the 4.2% expected during the placebo treatment of BD, whereas the rate of switch into mania after antidepressant drug treatment is 15–40% (58).

#### Usage in Bipolar Depression

BLT is a robust strategy treatment for bipolar depression and might be considered as a first line option for depressive episode in the course of BD (Table 1). Interestingly, BLT acts rapidly (within 4 days), has low side effects and low risk of manic switch in combination with mood stabilizing treatment (54). The most efficient light parameters are not yet fully determined, but it seems that patients are sensitive to light intensities below 10,000 lux, depending on the duration of exposure. Some observations suggest that depression could respond to light intensities as low as 300–500 lux (48, 50). Studies support both midday or morning BLT as an efficient therapy in BD depression and duration of light therapy could be increased progressively every week by 15 min to attain a target dose of 60 min per daily at 4 weeks (55). Furthermore, BLT should always be combined with a mood stabilizer, first to prevent manic switch in BD, but also to enhance and sustain the acute antidepressant effects of BLT (6).

### BLT for Sleep and Circadian Rhythm Abnormalities in Chronic Mood Disorders

#### BLT as an Effective Treatment in sleep and Circadian Rhythms Abnormalities

Light may act as a therapeutic mood stabilizer in patients with mood disorders during remission by stabilizing sleep alterations (like insomnia or longer sleep duration), and circadian rhythms (like evening chronotype or sleep phase delayed) (10). Indeed light exerts strong effects on mood through different pathways. First, light may affect mood through phase shifting of circadian rhythms (59–61). This circadian effect of light on mood results from its effect on the optimal alignment vs. misalignment of different circadian rhythms, which may be mediated through the phase shifting effects of light and modifying the duration of nocturnal melatonin secretion (59–61). This effect of light on mood via the circadian system is well-illustrated by SAD. SAD involves an internal misalignment of the circadian system and responds positively to treatments that resynchronize the biological clock, such as BLT. So, in addition to increasing the monoaminergic tone, application of BLT in the morning causes phase advance of endogenous circadian rhythms. These effects of light exposure on mood via modulation of the circadian system are not restricted to patients with SAD, but also in non-seasonal depression (both unipolar and bipolar), with available data that shows that BLT is an effective antidepressant or mood stabilizer (6).

Secondly, light exerts an effect on alertness, a parameter-influencing mood (62, 63). Impaired alertness is a common symptom in many affective disorders such as MDD, BD, or SAD (10). Increasing alertness has also been an effective strategy for improving mood (64–66). Light stimulates alertness through the synchronization of the homeostatic and circadian drives, and through melatonin suppression (10). And light has direct effect on alertness via the melanopsin retinal ganglion cells by impinging on the sleep-active neurons: through simultaneous inhibition of the sleep-inducing VLPO and activation of the monoaminergic arousal systems that are also involved in control of mood (10).

Thirdly, light may regulate mood disorders by improving sleep homeostasis with direct positive effect on the EEG delta sleep activity via the secretion of melanopsin, which is a photopigment expressed in a subset of retinal ganglion cells that activates the VLPO and improve by this way sleep homeostasis (10, 67, 68). This is again well-illustrated in studies with SAD patients that have a lower delta activity and lower sleep efficiency (69). Indeed, sleep deprivation combined with chronotherapeutics such as BLT have an antidepressant effect by improving sleep pressure (69).

**Figure 1:**
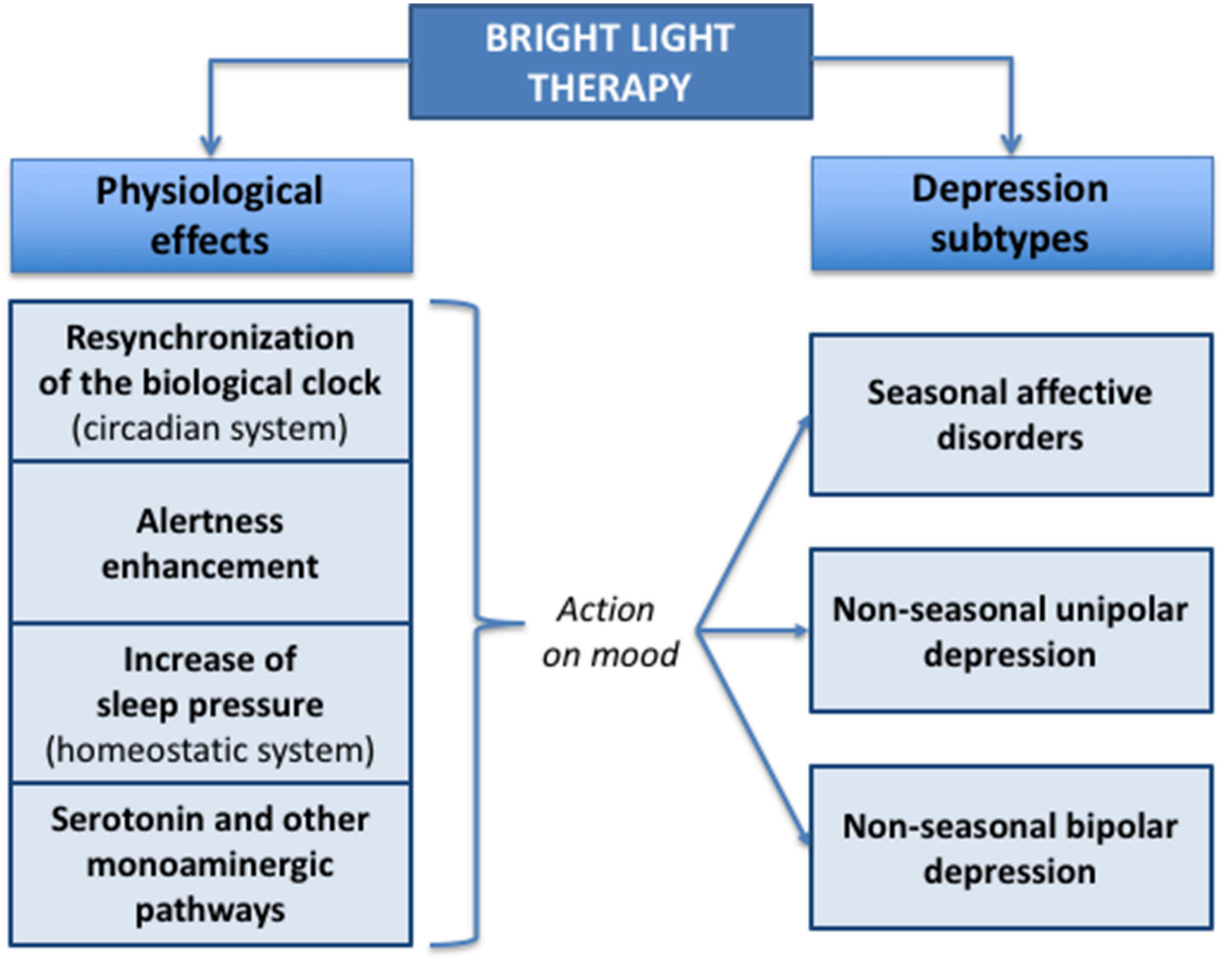
Effects of bright light therapy in mood disorders.

All these data support an impact of BLT by resynchronizing the biological clock (circadian system), and/or enhancing alertness, and/or increasing sleep pressure (homeostatic system), acting consequently on mood both in acute and remission phases (Figure 1).

#### Recommendations for Use in Patients With BD in Remission

Sleep and circadian rhythm abnormalities are frequent in mood disorders, even between acute episodes, contributing to poor functioning, and relapses (70, 71). For instance, in BD, studies of actigraphy and DMLO identify higher prevalence of delayed sleep phase (72) and exhibit greater variability in sleep duration, fragmentation, later, and more variable bedtimes than healthy controls (71). In this context, we suggest treating and applying recommendations for comorbid delayed phase sleep, insomnia, or hypersomnia in individuals suffering from mood disorders, even in remission. Recommending BLT in the context of such circadian and sleep comorbidities in mood disorders may thus be similar to recommendations for previous different subtype of depression detailed previously (Table 1).

## Discussion

This review draws up an inventory of scientific knowledge about the use of BLT in mood disorders thanks to a growing evidence about BLT evolving as an effective depression treatment not only to be used in SAD but also in non-seasonal depression and in BD. BLT is efficient on depression, acts rapidly, with low rates of manic switch, and can be easily prescribed combined or not with mood-stabilizing antidepressant, or other chronotherapeutic treatments such as sleep deprivation or lithium (6, 73). Table 1 summarizes different forms of BLT use in the different mood disorders subtypes. This review also highlights several shortcomings in the scientific literature, and paves the way for further studies to clarify the management of BLT across mood disorders by comparing different initiation protocols depending on depression subtypes, to validate its use as a maintenance treatment, and also to identify predictive biomarkers of response and tolerability.

First, there is a great heterogeneity of management of BLT across studies making it difficult to make recommendations for good practices of BLT (2, 11). For instance, whereas recent studies such as Lam et al. (40) that directly compared mono or adjunct therapies found larger effect of BLT/SSRI combination, some meta-analyses suggest on the other hand that monotherapy may be more effective (2, 11), maybe because of limitations previously advocated.

Secondly, where the lamp should be placed relative to the eye varies between available devices and across studies. Nevertheless, for most traditional BLT devices, it should be placed at eye level and at distance of 30–80 cm, in an adequately lighted room (where a newspaper may be read easily). The distance depends of the device and should be closely respected since Lux decreased quickly. Others new BLT devices should be placed as glasses and are easy to placed.

Thirdly, the vast majority of data are from review or meta-analyses in which the distinction is not made between unipolar disorder or BD, but also between seasonal characteristics or not, mostly because of lacking data and small sample sizes (55).

Nevertheless, our review aimed to propose global therapeutic strategies to use BLT in SAD, unipolar and bipolar disorders without seasonal characteristic and so, allows only partially personalizing BLT in mood disorder.

Because of the heterogeneity of study protocols and absence of comparisions between protocols, proper guidelines are difficult to define. In this context of absence of comparisons between protocols, we propose in BD to increase more slowly duration of exposure by 15 min every week to attain a target dose of 60 min per day at 4 weeks in case of insufficient response. In BD, a midday exposure might be safer with regards to manic switch, and should be preferred in cases where individuals have a history of antidepressant manic switch (74). BLT studies do not propose personalized BLT use modalities for bipolar disorder with seasonal pattern. For instance BLT studies assess only the effects of bright light therapy on SAD, which is a transdiagnostic disorder that may affect patients with unipolar and bipolar disorders, and proposed early morning administration with exposure duration of 30 min, using a light intensity of 10,000 lux.

Future studies will likely need to distinguish the use of BLT between bipolar disorder with seasonal pattern and unipolar disorder with seasonal pattern because patients with bipolar disorder with seasonal pattern are able to switch into mania (75). As patients with bipolar disorder without seasonal pattern, they are likely to need a midday utilization of BLT or a more progressive titration, and always in combination with an anti-manic treatment (75).

Finally, evidence suggests that light may play a role in the mood stabilizers therapeutic action by improving sleep quality, and stabilizing circadian rhythms (76). The role of sleep or circadian disturbances as disease course modifiers is well-established, mainly due to their association with treatment-refractory or prolonged mood phases and as predictors of early relapse (70). This review highlights the urgent need for good quality RCTs of BLT in BD during remission for relapses prevention through a potential improvement of sleep quality and stabilization of circadian rhythms.

## Conclusion

BLT should be considered as a stand-alone treatment option in patient with SAD, but also non-seasonal unipolar or bipolar depression. BLT in treating mood disorders is characterized by rapid and sustained effects both in mono- or adjunct-therapy, combined with antidepressant, or mood stabilizing drugs. However, the management of BLT needs to be clarified across mood disorders and future studies are expected to compare different dose-titration protocols, to validate its use as a maintenance treatment, and also to identify predictive biomarkers of response and tolerance. Finally, BLT may also be useful to improve sleep quality, decreased alertness, abnormalities in circadian rhythms such as sleep phase delay syndrome, that are frequently associated in mood disorders, in order to prevent mood early relapses and recurrences.

## Author Contributions

JM and PAG performed the literature search and analysis, and both contributed in the manuscript's redaction. JM wrote the first draft of the manuscript. PAG made the figure and the table.

### Conflict of Interest Statement

PG reports grants from Assistance Publique-Hôpitaux de Paris. The remaining author declares that the research was conducted in the absence of any commercial or financial relationships that could be construed as a potential conflict of interest.
